# Le contrôle de la taille de la famille au Maroc: une étude exploratoire qualitative auprès de femmes rurales

**DOI:** 10.48327/mtsi.v5i4.2025.667

**Published:** 2025-11-17

**Authors:** Chaimae MOUJAHID, Soufiane YASSARA, Jack E. TURMAN, Loubna AMAHDAR

**Affiliations:** 1 Laboratoire des sciences et ingénierie biomédicales, biophysique et santé, Institut supérieur des sciences de la santé, Université Hassan Premier, Settat 26000, Maroc; 2 Département des sciences sociales et comportementales, École de santé publique Richard M. Fairbanks, Université de l’Indiana, Indianapolis, IN 46202, États-Unis; 3 Département de pédiatrie, École de médecine, Université de l’Indiana, Indianapolis, IN 46202, États-Unis; 4 Faculté de médecine et de pharmacie, Université Mohammed V à Rabat, Maroc, Laboratoire de recherche en biotechnologie médicale (MEDBIOTECH), Département de pathologie, Institut national d’oncologie, Rabat, Maroc

**Keywords:** Contraception, Prise de décision, Rumeurs, Taille de la famille, Qualité de vie, Enfants, Maroc, Afrique du Nord, Contraception, Decision-making, Rumors, Family size, Quality of life, Children, Morocco, North Africa

## Abstract

**Objectif:**

Les femmes jouent un rôle central dans la planification familiale, mais leur capacité à exercer un contrôle sur leur fertilité est souvent influencée par des facteurs sociaux et relationnels. Cette étude explore les connaissances, attitudes et pratiques des femmes à l’égard de la planification familiale et des méthodes contraceptives actuellement disponibles, parmi la population des femmes mariées en âge de procréer vivant dans les zones rurales au Maroc.

**Méthodes:**

Nous avons mené des entretiens approfondis en face à face avec 49 femmes marocaines mariées dans une maternité rurale pendant leur période post-partum. À l’aide de questions ouvertes, nous avons examiné leurs pratiques contraceptives, leur autonomie décisionnelle et les influences sociales qui déterminent leurs choix en matière de procréation.

**Résultats:**

La plupart des participantes a déclaré utiliser des contraceptifs oraux, bien que nombre d’entre elles ait connu au moins une grossesse non désirée en raison d’une mauvaise utilisation des contraceptifs. Les femmes ont exprimé leur préférence pour des familles moins nombreuses afin d’assurer leur stabilité financière et une meilleure qualité de vie. Cependant, elles se heurtent souvent à la résistance de leur mari et de leur famille élargie lorsqu’elles tentent d’utiliser un moyen de contraception ou de limiter la taille de leur famille. Les idées fausses concernant les dispositifs intra-utérins (DIU) étaient très répandues, de nombreuses femmes exigeant le consentement de leur conjoint en raison de préoccupations liées à la santé sexuelle.

**Conclusion:**

Nos résultats soulignent le rôle important des facteurs sociaux et relationnels dans les choix des femmes en matière de procréation. Il est essentiel de s’attaquer aux idées fausses et d’améliorer l’accès à l’éducation en matière de santé génésique. L’implication des maris dans les séances de conseil peut renforcer les efforts de planification familiale et soutenir l’autonomie des femmes dans les décisions relatives à la fécondité.

## Abstract (en)

**Objective:**

Although women play a central role in family planning, their ability to control their fertility is often influenced by social and relational factors. This study explores the knowledge, attitudes and practices of married women of childbearing age living in rural areas of Morocco regarding family planning and the contraceptive methods currently available.

**Method:**

In-depth face-to-face interviews were conducted with 49 married Moroccan women in a rural maternity ward during their postpartum period. Using open-ended questions, we examined their contraceptive practices, decision-making autonomy and the social influences determining their reproductive choices.

**Results:**

Most participants reported using oral contraceptives, although many had experienced at least one unintended pregnancy due to contraceptive misuse. The women expressed a preference for smaller families in order to ensure financial stability and a better quality of life. However, they often encountered resistance from their husbands and extended families when attempting to use contraception or limit their family size. There were widespread misconceptions about intrauterine devices (IUDs), with many women requiring their spouse’s consent due to concerns about sexual health.

**Conclusion:**

Our findings highlight the important role of social and relational factors in women’s reproductive choices. Addressing misconceptions and improving access to reproductive health education is essential. Involving husbands in counselling sessions can strengthen family planning efforts and support women’s autonomy in fertility-related decisions.

## Introduction

Les programmes de planification familiale (PPF) qui encouragent l’utilisation de la contraception ont contribué à réduire les taux de fécondité dans de nombreux pays en développement [13]. Au Maroc, le PPF a été lancé en 1966 et a permis d’augmenter considérablement la couverture contraceptive tout en améliorant la santé maternelle et infantile [13]. Lutilisation des contraceptifs est un indicateur clé de la réussite de la mise en œuvre du planning familial. Elle constitue un facteur essentiel pour prévenir les grossesses non désirées, faciliter l’espacement des naissances, réduire les risques de mortalité infantile et limiter les avortements pratiqués dans des conditions dangereuses [4]. Au cours des deux dernières décennies, l’utilisation des contraceptifs par les femmes au Maroc a connu une augmentation significative. La prévalence contraceptive est passée de 9% en 1980 à 63% en 2004, puis à 67,4% en 2011 [13]. Selon l’étude nationale de 2018 sur la population et la santé familiale au Maroc, seulement 6,8% des femmes utilisaient le dispositif intra-utérin (stérilet; DIU) comme méthode contraceptive au moment de l’étude, malgré l’efficacité et le caractère réversible de ce mode de contraception. Néanmoins, ce taux d’utilisation du DIU variait en fonction des régions, plus élevé dans les régions urbaines (8,8%) que dans les zones rurales (3,8%). Dans 43% des cas d’utilisation du DIU, les femmes mentionnaient certaines préoccupations, notamment quant aux effets indésirables du DIU sur leur santé (32,6%), son influence sur les menstruations (9,4%) et le désaccord du mari (13,1%) [14].

Des efforts importants ont été déployés au niveau national pour améliorer l’accès aux méthodes contraceptives, renforcer la sensibilisation à la planification familiale et réduire les obstacles liés aux normes culturelles et aux connaissances des femmes. Des études antérieures ont identifié plusieurs facteurs influençant l’utilisation de la contraception au Maroc. Par exemple, une étude menée dans la province de Marrakech a révélé que le nombre d’enfants vivants et la mortalité infantile étaient des déterminants clés de l’utilisation de la contraception moderne, tandis que l’âge au mariage et l’âge de la femme étaient également des facteurs significatifs [16,22]. Par ailleurs, des recherches qualitatives ont mis en évidence que les normes de genre, telles que le refus parental, la stigmatisation et l’exclusion sociale des filles des services d’éducation à la santé sexuelle et reproductive, ainsi que le pouvoir décisionnel des membres de la famille concernant l’utilisation de la contraception, affectent l’accès aux services de santé sexuelle et reproductive dans la région de Marrakech-Safi [16].

Ces constats mettent en évidence la nécessité de renforcer les efforts visant à améliorer le recours des femmes aux méthodes contraceptives au Maroc. Ils soulignent également l’importance d’approfondir la compréhension des obstacles à l’adoption de certaines méthodes, en particulier le DIU, qui demeure l’une des options contraceptives les plus efficaces disponibles. À titre de comparaison, en Algérie, 47% des femmes mariées en âge de procréer qui n’avaient jamais utilisé de contraception ont cité l’opposition de leur mari/partenaire (12%) et la pression du partenaire (4%) comme raisons de ne pas utiliser de contraception. Les normes religieuses et culturelles traditionnelles, ainsi que les obligations légales, placent souvent les hommes à la tête de leurs femmes et de leurs familles [2].

L’objectif principal de cette étude était d’explorer les connaissances, attitudes et pratiques des femmes à l’égard de la planification familiale et des méthodes contraceptives actuellement disponibles, parmi la population des femmes mariées en âge de procréer vivant dans les zones rurales au Maroc. En outre, nous avons voulu étudier l’influence des normes de genre sur la prise de décision en matière de planification familiale et identifier les facteurs facilitant ou limitant l’adoption des méthodes contraceptives, en particulier les méthodes de longue durée (le DIU).

## Matériels et méthodes

Les données ont été recueillies dans les services de maternité de quatre hôpitaux provinciaux situés dans différents districts du Maroc:

l’hôpital provincial Hassan II à Settat (au nord-ouest);
l’hôpital provincial Moulay Abdellah à Salé (au nord-ouest également);
l’hôpital Sidi Mohamed ben Abdellah à Essaouira (au sud-ouest);
l’hôpital Ibno Baja à Taza (au nord-est).

Ces hôpitaux provinciaux desservent à la fois des zones urbaines et rurales dans leurs provinces respectives, ce qui garantit une représentation diversifiée des participants à l’étude.

Cette étude a utilisé une approche ethnographique pour explorer la compréhension qu’ont les femmes marocaines de la planification familiale, leurs attitudes à l’égard des méthodes contraceptives et les obstacles qu’elles rencontrent dans l’accès à la contraception. La collecte des données s’est faite par le biais d’entretiens semi-structurés à questions ouvertes, en suivant un processus d’analyse itératif.

L’étude ciblait les femmes mariées en âge de procréer, récemment accouchées et résidant dans les zones rurales des provinces sélectionnées, souvent confrontées à des contraintes socio-économiques telles que des niveaux d’éducation faibles et un accès limité aux services de santé. Les participantes ont été recrutées au cours de leur séjour *post-partum* dans les maternités, parmi toutes celles répondant aux critères d’inclusion et ayant consenti à participer à l’étude.

Une stratégie d’échantillonnage non probabiliste a été utilisée, combinant l’échantillonnage de commodité et l’échantillonnage au jugé dans la phase initiale [5,11,17]. Une analyse préliminaire des premiers entretiens a permis de vérifier la pertinence des participantes et d’affiner les critères de sélection, conduisant à un échantillon final de 49 personnes. La saturation des données a déterminé la taille finale de l’échantillon, le recrutement s’arrêtant lorsqu’aucun nouveau thème n’émergeait des récits des participants. La collecte des données s’est déroulée sur huit mois, de septembre 2020 à avril 2021. Le processus de recherche a commencé par des essais pilotes du guide d’entretien approfondi avec des femmes partageant des caractéristiques similaires, afin d’affiner les questions de recherche et d’identifier les lacunes terminologiques. Tout au long de l’étude, les entretiens approfondis ont été façonnés par les récits reproductifs des participantes, favorisant la relation et l’engagement entre les chercheurs et les personnes interrogées. Certaines participantes ont demandé des entretiens de suivi, ce qui a donné lieu à des réunions supplémentaires. Les entretiens ont duré entre 30 minutes et 2 heures, avec une durée moyenne de 1 heure 30.

Des entretiens approfondis ont été menés sur la base d’un guide semi-structuré, élaboré en s’appuyant sur une analyse documentaire concernant les déterminants sociaux de la planification familiale et de la santé reproductive dans les pays du Maghreb, ainsi que sur les instruments de recherche qualitative employés dans des études analogues. Par la suite, une adaptation au contexte local a été réalisée, impliquant un processus de discussion entre les auteurs, et prenant en considération les spécificités culturelles et linguistiques des femmes résidant en milieu rural au Maroc. Le guide comprenait essentiellement des questions ouvertes visant à examiner les connaissances, les attitudes et les pratiques relatives à la planification familiale, ainsi qu’à étudier l’influence des normes de genre et les obstacles et facteurs favorisant l’adoption de la contraception (Annexe 1), permettant aux participantes de partager leurs expériences avec leurs propres mots. Tous les entretiens ont fait l’objet d’un enregistrement audio, puis d’une transcription pour analyse.

Les enregistrements audios ont été transcrits et les verbatims ont été traduits du dialecte marocain vers l’anglais. L’analyse thématique a été utilisée, en commençant par la génération de codes initiaux, qui ont ensuite été regroupés en thèmes potentiels. Les chercheurs ont relu les transcriptions plusieurs fois pour affiner les thèmes émergents. Le codage et l’organisation des données ont été effectués à l’aide du logiciel MAXQDA 20, complété par une analyse manuelle dans la phase initiale.

L’étude a reçu l’approbation éthique du Comité d’éthique pour la recherche biomédicale de la Faculté de médecine et de pharmacie de Rabat (CERB: 45/21). Les participantes ont donné leur consentement éclairé avant de prendre part à l’étude, garantissant ainsi leur participation volontaire et la confidentialité.

## Résultats

Le Tableau I résume les caractéristiques sociodémographiques des femmes ayant participé à l’étude. Nos données qualitatives mettent en évidence cinq thèmes significatifs relatifs aux connaissances, attitudes et pratiques (CAP) des participantes en matière de planification familiale, à savoir:

la prise de décision en matière de planification familiale;
l’utilisation actuelle de la contraception et les comportements à l’égard des méthodes de contraception;
la taille de la famille des femmes et le concept de famille;
les connaissances des femmes en matière de planification familiale et de méthodes contraceptives;
les barrières affectant l’utilisation des contraceptifs, en particulier les stérilets.

**Tableau I:** Caractéristiques des participantes

Province	Nombre d’entretiens individuels	Tranche d’âge (en années)	Nombre moyen d’enfants	Nombre de femmes au foyer	Niveau d’éducation
Taza	12	(24-44)	3	12	Non scolarisées = 4 Primaire = 6 Collège = 1 Lycée = 1
Essaouira	13	(19-37)	3	13	Non scolarisées = 4 Primaire = 8 Collège = 1
Salé	11	(20-38)	2,81	10	Non scolarisées = 2 Primaire = 4 Collège = 1 Lycée = 4
Settat	13	(17-36)	2,53	12	Non scolarisées = 4 Primaire = 4 Collège = 4 Lycée = 1

Le genre joue un rôle crucial dans la détermination de la personne qui a le dernier mot sur l’utilisation par un couple d’une méthode de contraception.

Certaines femmes subissent une pression supplémentaire de la part de leur mari, en particulier dans la quête d’un fils. L’une des personnes interrogées a fait part de son expérience: « *J’ai déjà quatre filles et mon mari veut un fils. Je ne peux plus le supporter. Outre les difficultés que j’ai rencontrées pendant la grossesse et l’accouchement à cause de ma maladie cardio-vasculaire, toute la famille attend un fils avec impatience ».* (Aicha, Essaouira)

Pour la plupart des personnes interrogées, les décisions en matière de contrôle des naissances sont une affaire commune, et le choix d’une méthode contraceptive, en particulier au-delà des contraceptifs oraux, requiert souvent l’approbation du mari. Parfois, cela va jusqu’à une forte résistance à l’utilisation de DIU. Comme l’a expliqué une participante: « *Il voulait un troisième enfant, mais j’ai refusé parce que je ne voulais pas d’une autre grossesse. J’ai suggéré d’utiliser le stérilet, mais il a refusé, car il souhaite toujours avoir d’autres enfants »* (Marzouka, Taza). Les données relatives à la prise de décision en matière de planification familiale et de contraception moderne montrent que les maris et les beaux-parents influencent considérablement le moment, la manière et même l’opportunité de recourir à la planification familiale et d’utiliser la contraception.

En outre, les belles-mères exercent également une autorité considérable en matière de contrôle des naissances. Elles influencent la femme de leur fils et, lorsqu’elles souhaitent agrandir la famille, elles persuadent souvent la femme de ne pas utiliser de méthodes contraceptives et peuvent même lui interdire l’accès aux contraceptifs dans les centres de santé. Une femme a déclaré: « *Mon mari et sa famille préfèrent les enfants de sexe masculin et souhaitent agrandir la famille. Cependant, je n’ai aucun désir d’avoir plus d’enfants »* (Randa, Taza). Une autre participante a déclaré: « *Mon conjoint et sa famille favorisent fortement les fils, et maintenant que j’ai subi une ligature des trompes… mais c’est après avoir eu cinq enfants »* (Marzouka, Taza).

Les femmes ont souligné qu’elles sont souvent contraintes de recourir à la planification familiale en raison des conséquences personnelles et physiques des grossesses. « *Je ne veux pas avoir d’autres enfants; je suis satisfaite de mes enfants actuels; j’ai enduré des épreuves importantes pendant la grossesse, ce qui a affecté ma santé et m’a laissée épuisée »* (Randa, Taza).

À l’exception des femmes primipares qui adhéraient à la croyance normative selon laquelle le but premier du mariage était la procréation et que la grossesse devait suivre rapidement le mariage, presque toutes les participantes utilisaient au moins une forme de contraception. Bien que les méthodes de contraception soient légalement accessibles à toutes les femmes mariées, celles qui n’ont jamais eu d’enfant y ont rarement recours. Aucune des nouvelles mères n’avait utilisé de méthode contraceptive moderne pour réguler sa fécondité avant la naissance de son premier enfant. La pilule contraceptive est apparue comme la méthode la plus répandue parmi nos participantes. Les femmes multipares, en particulier, ont expérimenté diverses méthodes contraceptives. Certaines se sont plaintes des effets indésirables, affirmant que ces méthodes présentaient des risques pour la santé. Une participante a fait part de son expérience: « *J’utilise la pilule comme moyen de contraception pour espacer mes grossesses, mais elle a eu des effets indésirables. J’ai développé un fibrome à cause de la pilule et cela m’a conduit à deux avortements »* (Rachida, Taza). Malgré les effets indésirables et le risque d’oubli, toutes les femmes ont exprimé leur confiance en la pilule. Pour les femmes qui souhaitaient avoir plusieurs enfants, la méthode conventionnelle était l’abstinence périodique, ce qui a soulevé des questions quant à la nécessité d’une contraception moderne. Une autre participante a déclaré: « *J’utilise la pilule depuis cinq ans, depuis la naissance de mon premier enfant. C’est efficace et j’ai confiance en cette méthode. J’appréhende les petites interventions chirurgicales (le DIU). Les gens en parlent souvent de façon négative » (Ghizlan, Essaouira).*

Peu de femmes ont opté pour le DIU comme méthode contraceptive, mais celles qui l’ont fait l’ont fait avec succès et ont exprimé le désir de continuer à l’utiliser pour l’espacement ou la limitation des naissances. Leur motivation provenait des expériences positives de leurs mères, de leurs sœurs et de leurs amies. Une participante a déclaré: « *Je me suis récemment fait poser un stérilet. Je l’avais utilisé pendant sept ans avant d’avoir cet enfant, et je l’ai retiré pour concevoir. J’ai l’intention de revenir à cette méthode contraceptive parce que j’ai trouvé que la pilule affectait négativement mon équilibre hormonal. Avec le stérilet, j’ai pu porter des objets lourds sans ressentir d’effets indésirables. Son utilisation dépend de l’état de l’utérus de la femme. Une femme dont l’utérus est sain et propre peut s’acquitter de ses tâches ménagères et soulever des objets lourds sans problème. L’épouse de mon beau-frère a porté un stérilet pendant 12 ans* » (Aicha, Essaouira). Toutes les participantes ont exprimé le souhait de limiter le nombre d’enfants dans leur famille à un nombre idéal de deux enfants de sexe différent, certaines mentionnant que leur nombre idéal était de trois enfants.

Toutefois, la plupart des femmes ayant plus de deux enfants a déclaré avoir dépassé ce nombre idéal en raison du désir d’avoir une fille et un garçon ou de l’insistance de leur mari et de leur famille. Un autre facteur important pour avoir plus d’enfants est la préférence pour les fils. Ainsi, une participante a déclaré: « *Mon mari désire un fils, et j’ai déjà deux filles. Mais je ne souhaite pas avoir plus d’enfants car j’ai connu des difficultés pendant la grossesse et l’accouchement. Les hommes, eux, préfèrent souvent avoir un fils »»* (Kawtar, Salé).

Plusieurs femmes interrogées ont souligné l’importance de la présence d’un fils dans le foyer pour l’éducation de leurs filles. Elles pensaient qu’un fils protégerait ses sœurs des menaces extérieures et les accompagnerait à l’école, garantissant ainsi leur sécurité en l’absence du père. Une participante a expliqué: « *Ma belle-mère souhaite une famille nombreuse; elle prie pour avoir cinq filles ou six fils. Mon mari pense qu’il est préférable d’avoir d’abord un fils, car il peut contribuer à l’éducation de ses sœurs. La fille devrait être la première à prendre ses propres décisions sans être soumise à l’autorité de son frère. Dans notre communauté, lorsqu’un garçon naît en premier, il a souvent l’autorité sur sa sœur. Mon frère aîné peut faire ce qu’il veut »* (Khadija, Settat). Dans ce contexte, certaines personnes questionnées préféraient que le dernier enfant soit un garçon afin d’épargner à leurs filles une double pression, l’une de la part de leur père et l’autre de la part de leur frère.

Les personnes interrogées ont souvent souligné la complexité croissante de la vie actuelle lorsqu’on leur a demandé si elles voulaient d’autres enfants ou si elles abordaient la question de la fertilité et de la parentalité. « *Je ne souhaite plus avoir d’enfants; actuellement, élever des enfants est plus exigeant. Il faut subvenir à leurs besoins financiers, ce qui n’est pas facile. Aujourd’hui, les enfants nous demandent pourquoi nous les avons mis au monde si nous ne pouvons pas subvenir à leurs besoins »* (Rachida, Essaouira).

Les femmes interrogées ont révélé que cette génération nécessite des ressources financières importantes et que la situation financière actuelle ne permet souvent d’élever qu’un ou deux enfants. Elles ont insisté sur les contraintes physiques et financières liées au fait d’avoir des enfants, qui dépassent parfois leurs moyens. Une participante a déclaré: « *Mon mari veut quatre enfants, mais notre situation socio-économique ne nous le permet pas. Pour élever des enfants, il faut de l’argent, de l’éducation et des vêtements appropriés. Il ne s’agit pas seulement d’avoir un enfant, mais de s’assurer qu’il a de la nourriture et des vêtements. Que nous ayons deux ou trois enfants, ils doivent avoir une bonne vie, des vêtements appropriés et une éducation »* (Nezha, Taza).

Le concept de famille varie d’une participante à l’autre. Pour la plupart des femmes, la famille est considérée comme la pierre angulaire de la société, et les hommes comme les femmes aspirent à fonder une famille. « *La famille est le destin de chaque individu; tout le monde souhaite fonder une famille, et ne pas avoir d’enfants est un défi. Un jour, je compterai sur mes enfants pour s’occuper de moi quand je serai âgée »* (Hafida, Essaouira). Pour ces femmes, une famille se compose d’une mère, d’un père et d’au moins un enfant. Le rôle des parents est de veiller au bien-être, à l’habillement et à l’éducation de leurs enfants. Les responsabilités du père consistent à assurer la sécurité, la subsistance et le logement de la famille, tandis que la femme s’occupe de ses enfants et de son mari, chacun dans son rôle. Les femmes ont indiqué que les enfants de la famille obéissaient aux instructions de leurs parents et allaient à l’école. Certaines ont également indiqué que les enfants étaient considérés comme un projet d’investissement et qu’en grandissant, ils étaient censés apporter un soutien financier à leurs parents et rembourser les soins qu’ils avaient reçus pendant leur enfance. Une participante a expliqué: « *Nous mettons les enfants au monde pour les éduquer. Lorsqu’ils grandissent, ils doivent nous soutenir financièrement et se tenir à nos côtés »* (Es Zahra, Taza).

Les femmes de toutes les régions ont pu citer plusieurs méthodes de planification familiale, en particulier certaines méthodes modernes. Toutefois, leurs connaissances restaient limitées concernant le fonctionnement, l’efficacité et les modalités d’utilisation de chaque méthode. En dehors des méthodes les plus connues, comme les pilules et les contraceptifs injectables, les participantes avaient peu d’informations sur le DIU, la ligature des trompes, les implants ou la vasectomie. Par ailleurs, elles mentionnaient aussi l’allaitement maternel comme une méthode naturelle pour éviter les grossesses parmi les approches traditionnelles.

Les participantes de toutes les régions ciblées ont souligné l’importance de la planification familiale pour la santé maternelle et infantile et le bien-être général de la famille.

Presque toutes les participantes à l’étude ont connu au moins une grossesse non désirée, principalement en raison d’une mauvaise utilisation de la contraception, en particulier de la pilule, de l’incapacité du mari à s’abstenir lorsqu’il pratique l’abstinence périodique comme méthode naturelle de contraception, ou de la pression familiale pour avoir plusieurs enfants. Dans certains cas, les maris interviennent dans la gestion de la contraception. Par exemple, l’une des participantes a expliqué: « *Mon mari a géré une grossesse non désirée, car il m’a rapporté une pilule micro-progestative au lieu d’une oestro-progestative; je l’ai prise et je suis tombée enceinte… »* (Fatiha, Salé). Cependant, il est préoccupant de constater que les femmes ont une connaissance limitée de l’efficacité de la contraception, en particulier de la pilule (comment la prendre, comment l’utiliser correctement et que faire en cas d’oubli d’une dose). Presque toutes les femmes ont connu au moins une grossesse non désirée à cause d’une mauvaise utilisation de la pilule, souvent attribuée à un manque d’information sur cette méthode contraceptive largement utilisée au Maroc.

Ainsi, une participante a déclaré: « *J’ai cessé d’utiliser la pilule contraceptive lorsque mon mari était absent. À son retour, j’ai repris la pilule la veille de son arrivée, mais je suis quand même tombée enceinte. Je pense que je ne l’ai peut-être pas prise correctement. Je connais les injections contraceptives, mais j’hésite à les utiliser en raison des risques d’ostéoporose. De plus, j’appréhende une intervention chirurgicale mineure (stérilet) qui peut attraper le pénis de l’homme lors d’un rapport sexuel »» (Bouchra, Essaouira).*

Dans toutes les régions, la plupart des femmes considèrent la famille et les amis comme les sources d’information les plus fiables et les plus immédiates en matière de contraception. Elles étaient fortement influencées par les expériences de leur famille et de leurs amies, l’adoption d’une méthode contraceptive faisant souvent suite à une expérience positive rapportée par un membre de la famille, en particulier pour les méthodes telles que le stérilet et la pilule.

Une participante a exprimé cette influence en déclarant: « *Les méthodes contraceptives que je connais, même si je ne les utilise pas, comme le stérilet ‘Amalia Sghira’ (petite chirurgie), j’ai entendu des femmes qui l’ont utilisé dire qu’il était efficace et bénéfique. Ma sœur l’a porté pendant sept ans et j’envisage de l’utiliser après mon prochain accouchement. Mes voisins ont mentionné qu’une femme était tombée enceinte avec le stérilet, mais c’est parce qu’elle portait des objets lourds. J’ai aussi quelques connaissances sur les injections »»* (Samira, Essaouira).

Peu de femmes savaient que le stérilet était une méthode de contraception. Le terme local « *Amalia Sghira »,* qui signifie « petite chirurgie », a suscité des impressions négatives et des inquiétudes à propos de cette méthode, car beaucoup pensaient à tort qu’il s’agissait d’une intervention chirurgicale.

Une participante a fait part de son appréhension en déclarant: «*… Je connais l’existence d’Amalia Sghira, mais j’ai peur parce que je ne la comprends pas très bien. Je pensais qu’il fallait des ciseaux et du matériel chirurgical. Le terme « petite chirurgie » m’a fait peur. Un membre de ma famille m’a parlé de sa « petite chirurgie », ce qui m’a effrayée car je pensais qu’il s’agissait d’une intervention chirurgicale. Je veux espacer mes naissances et attendre cinq ans avant d’avoir un deuxième enfant »* (Khadija, Settat).

Une autre femme a fait part de son expérience: « *J’ai utilisé ‘Amalia Sghira’ pendant trois ans, mais je l’ai fait enlever parce qu’il provoquait des saignements excessifs. Je n’ai pas pu jeûner pendant tout le mois de Ramadan à cause de cela* » (Fatiha, Taza).

Les mythes qui entourent le DIU, comme la croyance qu’il peut « se déplacer dans l’abdomen, entraînant une intervention chirurgicale », ont été mentionnés comme des obstacles à son utilisation. Même lorsque leurs conjoints envisagent d’utiliser le stérilet, la plupart des femmes s’inquiètent du fait que cette méthode limite leur capacité à accomplir les tâches ménagères.

Une femme a déclaré: « *Le stérilet peut migrer vers le dos ou une artère. Un membre de la famille m’a dit qu’elle avait commencé à avoir mal au dos et qu’elle soupçonnait que le stérilet s’était déplacé dans son dos, alors elle l’a fait retirer »»* (Ghizlan, Essaouira).

Certaines femmes ont également mentionné l’insensibilité de leur mari à l’impact potentiel de la pilule sur leur santé. Les maris refusent souvent d’utiliser des préservatifs parce qu’ils craignent que cela ait un effet sur leur virilité, le planning familial étant généralement perçu comme une responsabilité féminine. « *Il a refusé d’utiliser des préservatifs, affirmant que c’est à moi, en tant que femme, qu’il incombe de gérer le planning familial »,* a fait remarquer Kawtar de Settat.

L’indifférence des prestataires de soins, les longs délais d’attente dans les établissements de santé et l’indisponibilité de certains services, comme la pose de stérilets dans les établissements de soins de santé primaires, dissuadent les femmes de chercher activement à obtenir des conseils en matière de contraception.

Les restrictions strictes imposées à la mobilité des femmes sont apparues comme un obstacle important dans toutes les régions. Les femmes ne peuvent quitter leur domicile sans l’accord de leur mari ou de leur belle-mère. Celles qui s’aventurent seules, même pour des raisons médicales, sont considérées comme manquant de respect envers leur famille.

Une participante a décrit son expérience en disant: « *Mon mari m’a apporté la pilule de la pharmacie et je n’ai pas le droit de quitter la maison sans sa permission. J’ai fini par tomber enceinte. Cette pilule spécifique, de type ‘progestatif’, est la raison de ma grossesse. Au début, j’ai résisté parce qu’il changeait la pilule pour moi. Le centre de santé où je pouvais obtenir la bonne pilule est loin de chez nous, et il est difficile d’organiser le transport et d’autres aspects logistiques. De plus, l’infrastructure n’est pas fiable, ce qui complique encore les choses »* (Fatiha, Sale).

## Discussion

L’amélioration de la santé reproductive au Maroc est depuis longtemps un défi persistant [15], et la planification familiale offre un potentiel considérable pour atteindre cet objectif [10]. Cependant, peu de recherches ont été menées sur les connaissances, attitudes et pratiques des femmes marocaines en matière de planification familiale.

Les narrations des participantes attestent de l’incidence significative des normes sociétales, des anticipations familiales et des vécus sur la compréhension et la prise de décision relatives à la planification familiale au Maroc. Cela correspond à des recherches récentes menées dans d’autres pays à faible revenu [7,8, 20]. Les résultats obtenus indiquent que l’autonomie reproductive des femmes, les perceptions culturelles relatives à la reproduction et les pressions exercées par les réseaux sociaux interagissent de manière concertée pour influencer le recours à la contraception, agissant tantôt comme un facteur facilitateur, tantôt comme une contrainte. Ces constatations confirment les conclusions de recherches effectuées dans d’autres pays à faible revenu [18].

Au Maroc, l’utilisation de la contraception n’est pas considérée comme normale avant la première conception, ce qui amène les femmes et les couples à envisager la planification familiale après la période du *post-partum.*

Le désir de fonder une famille après le mariage est un thème commun à toutes les participantes. Cela met en évidence la croyance sociale largement répandue selon laquelle un partenariat réussi implique d’avoir des enfants. Cette notion est étroitement liée à l’importance de la maternité en tant que moyen pour les femmes d’accomplir leur destin et de s’assurer un rôle prépondérant dans la construction sociétale d’une féminité idéalisée [21].

Ces normes culturelles dominantes influencent fortement la prise de décision de nos participantes concernant la planification familiale après le mariage. Cependant, les discussions et négociations réelles ont eu lieu dans le cadre de leur partenariat conjugal.

L’absence d’enfant de sexe masculin au sein du ménage conduit souvent à une augmentation du nombre total de naissances, ce qui contribue à expliquer les variations observées dans la taille des familles. Du point de vue des femmes, les hommes préfèrent avoir des enfants de sexe masculin parmi leur progéniture. Cette conclusion implique que, selon eux, les fils sont les protecteurs de la famille en l’absence du père. Elle est comparable à une étude qualitative menée en Éthiopie, qui a également révélé une préférence pour les garçons par rapport aux filles. En outre, le fait d’avoir une famille nombreuse est un symbole de statut et une marque de popularité dans d’autres communautés [8].

Dans notre étude, les femmes exprimaient une préférence pour les familles de petite taille, invoquant principalement des contraintes socioéconomiques. Ce constat est cohérent avec la littérature où plusieurs enquêtes nationales et comparatives montrent une réduction progressive de la taille idéale de la famille au Maroc et dans des contextes similaires. Par exemple, la taille familiale souhaitée est passée de 5 à 3,7 enfants entre 1980 et 1995 au Maroc, tandis qu’une baisse significative de la fécondité a été observée en milieu rural (de 6,6 à 3 enfants par femme) en lien avec l’éducation et l’emploi des femmes [3,6]. Ces résultats confirment que la préférence pour de plus petites familles s’inscrit dans un processus plus large de transition démographique et de transformation socio-culturelle. Cependant, il n’est pas rare que les maris souhaitent des familles plus nombreuses. Comme pour les autres décisions en matière de reproduction, les choix des femmes concernant la taille de la famille font l’objet de discussions permanentes avec leurs maris et impliquent parfois la belle-mère, qui joue un rôle important dans la dynamique familiale marocaine.

La plupart des femmes interrogées dans le cadre de notre enquête, en particulier les primo-mères ou celles qui n’ont jamais utilisé de contraception biomédicale, ont une connaissance limitée des méthodes contraceptives, la pilule étant le choix le plus populaire. De même, leur compréhension de la Méthode de l’allaitement maternel et de l’aménorrhée était limitée, comme l’ont montré d’autres recherches récentes [21].

Notre étude montre qu’en raison d’une mauvaise utilisation des contraceptifs, un nombre significatif de femmes sont exposées au risque de grossesses non désirées. Nos résultats révèlent plusieurs mythes et obstacles associés à l’utilisation de diverses méthodes contraceptives, en particulier le stérilet. Un nombre important de femmes ont des idées fausses et des inquiétudes à propos de cette méthode, principalement basée sur des informations provenant d’amies, de membres de la famille et de conjoints. Les expériences personnelles et les anecdotes provenant des réseaux sociaux ont eu une influence plus importante sur la perception de la sécurité que les conseils médicaux. Des résultats similaires ont été rapportés dans d’autres études où l’acceptation du DIU était facilitée par les conseils complets des prestataires de soins de santé, mais entravée par la peur et le rejet des amies, de la famille et des voisins [9,18]. Selon les résultats de notre étude, la plupart des femmes souhaitent des contraceptifs faciles à utiliser, sans complications et offrant la liberté de choisir, d’utiliser et d’accéder à la méthode contraceptive la plus appropriée sans être limitées par des facteurs socio-économiques, tels que les difficultés d’accès aux services de santé ou la nécessité d’obtenir l’autorisation de leur conjoint. Le manque de communication entre le personnel de santé et les femmes dans les unités de planification familiale des établissements de santé, ainsi que l’absence d’options contraceptives spécifiques dans les régions rurales, notamment la pose de DIU, ont un impact négatif sur la sensibilisation et l’accès des femmes aux méthodes de contrôle des naissances. Il est donc impératif d’aborder ces questions en fournissant des informations complètes, en encourageant une approche amicale, en organisant des séances de conseil et en impliquant les maris pour dissiper les fausses rumeurs sur les contraceptifs.

Pour répondre à ces défis, plusieurs interventions ont démontré leur efficacité dans des contextes similaires [1,12,19,23].

**Programmes de sensibilisation communautaire** Des initiatives telles que les visites à domicile de motivation systématique (VDMS) ont permis de renforcer l’information sur la contraception et de favoriser son acceptation dans les communautés rurales. Ces programmes, en impliquant les leaders communautaires et en adaptant les messages aux réalités locales, ont contribué à une meilleure compréhension et utilisation des méthodes contraceptives.
**Renforcement de l’autonomisation des femmes** L’autonomisation des femmes est un facteur clé pour améliorer leur santé reproductive. Des programmes visant à renforcer les compétences des femmes, à promouvoir leur participation à la prise de décision et à améliorer leur accès à l’éducation et à l’emploi ont montré des résultats positifs en matière de santé reproductive.
**Amélioration de l’accès aux services de santé reproductive** L’extension des services de santé reproductive, notamment par la formation de prestataires de santé communautaires et l’utilisation de la télémédecine, a permis d’améliorer l’accès aux services contraceptifs dans les zones reculées. Ces approches ont facilité la diffusion de l’information et l’accès aux méthodes contraceptives.
**Éducation sexuelle complète** Bien que confrontée à des obstacles culturels, l’éducation sexuelle complète a montré son efficacité pour réduire les comportements à risque et augmenter l’utilisation des contraceptifs. Son intégration dans les programmes scolaires et communautaires, avec la formation des enseignants et l’implication des parents, est essentielle pour son succès.

Par conséquent, une approche intégrée combinant sensibilisation communautaire, autonomisation des femmes, amélioration de l’accès aux services de santé reproductive et éducation sexuelle complète est cruciale pour répondre aux besoins non satisfaits en matière de contraception et améliorer la santé reproductive des femmes en milieu rural marocain.

## Conclusion

Notre étude met en lumière les obstacles posés par diverses normes de genre à l’utilisation de certains moyens de contraceptions. Les décisions de planification familiale des femmes que nous avons étudiées sont directement influencées ou complétées par des stéréotypes de genre pertinents. Nos résultats révèlent que les décisions en matière de planification familiale sont influencées par plusieurs facteurs socio-culturels et économiques, notamment le pouvoir décisionnel du mari, la nécessité de son consentement, la préférence pour une descendance masculine, le statut inférieur des femmes, leurs connaissances limitées en contraception, ainsi que l’influence de la famille élargie. Malgré ces contraintes, la plupart des femmes souhaitent une méthode contraceptive sûre et réversible. Le statut socio-économique apparaît déterminant: les couples aspirent à une meilleure qualité de vie pour leurs enfants et associent la limitation des naissances à cette perspective, bien que, dans les ménages défavorisés, la réduction du nombre d’enfants demeure difficilement envisageable.

Ces aspects sont interconnectés et donnent lieu à des considérations complexes qui se traduisent souvent par des conflits ou des préférences divergentes parmi les couples mariés en matière de planification familiale. La participation des femmes à la prise de décision est souvent limitée. Beaucoup d’entre elles adoptent les préférences de leur mari en raison du contrôle exercé par l’homme dans le ménage et des circonstances connexes.

Pour résoudre ces problèmes, il est impératif de dissiper les idées fausses et les craintes entourant les DIU et d’améliorer le comportement de soutien des professionnels de la santé. Par conséquent, des interventions sont nécessaires pour renforcer l’autonomie des femmes en matière de prise de décision et leurs capacités de négociation dans les questions liées à la planification familiale.

Il est essentiel de donner la priorité à la disponibilité des traitements, tels que les contraceptifs injectables et la contraception définitive qui nécessitent l’acceptation du partenaire. L’éducation des filles et l’implication des hommes dans la planification familiale peuvent faciliter les décisions à long terme en matière de santé reproductive et améliorer la compréhension des différentes techniques contraceptives chez les femmes.

## Déclaration du Comité d’éthique institutionnel

Létude a été menée conformément à la Déclaration d’Helsinki et approuvée par le Comité d’éthique de la Faculté de médecine et de pharmacie de Rabat, Maroc (référencé sous CERB: 45/21)

## Déclaration de consentement éclairé

Un consentement éclairé a été obtenu auprès de toutes les participantes impliquées dans l’étude.

## Financement

Cette recherche n’a reçu aucun financement externe.

## Contributions des auteurs et autrices

Conceptualisation: CM et JETJ; Méthodologie: CM, JETJ et LA; Analyse formelle: CM et JETJ; Investigation: CM; Curation des données: CM et LA; Rédaction-préparation du brouillon original: CM SY et JETJ; Rédaction-révision et édition: CM, SY, JETJ et LA; Visualisation: JETJ et LA; Supervision: LA. Tous les auteurs ont lu et approuvé la version publiée du manuscrit.

## Déclaration de liens d’intérêt

Aucun lien d’intérêt n’a été déclaré.

## Annexe: Fiche d’information sur l’étude pour évaluer les connaissances et attitudes a l’égard des résultats en matière de santé maternelle au Maroc

Appendix: Fact sheet on the study to assess knowledge and attitudes toward maternal health outcomes in Morocco

### FICHE D’INFORMATION SUR L’ÉTUDE POUR EVALUER LES CONNAISSANCES ET ATTITUDES A L’EGARD DES RESULTATS EN MATIERE DE SANTE MATERNELLE AU MAROC

**Partie de planification familiale et taille de famille: Perception, connaissances et prise de décision.**

**1. Caractéristiques Sociodémographiques**

- Quel est votre âge?
- Quel est votre statut matrimonial actuel? (Célibataire, mariée, en union libre, divorcée, veuve, etc.)
- Combien d’enfants avez-vous actuellement?
- Quel est le niveau d’études le plus élevé que vous avez atteint?
- Quelle est votre activité professionnelle actuelle?
- Vivez-vous en milieu urbain, périurbain ou rural?
- Quelle est votre religion (si vous souhaitez répondre)?
- Quel est votre âge au premier mariage?
- Quel est le niveau d’étude de ton mari?
- Quel est la profession de votre conjoint?
- Est-ce qu’il a une deuxième femme ou plus que vous? Si plus combien?
- Vivez-vous ensemble au sein de même ménage ou séparé?

**2. La prise de décision en matière de planification familiale**

- Pouvez-vous me décrire comment se prennent les décisions liées à la planification familiale dans votre couple ou votre foyer? qui prend la décision? qui autre?
- Quelles sont, selon vous, les principales influences sur vos décisions concernant la planification familiale? comment?
- Quel rôle pensez-vous que votre partenaire joue dans le processus de décision concernant l’utilisation de méthodes contraceptives?
- Selon toi, le mari va-t-il également une responsabilité dans la gestion de la contraception?
- Est-il généralement d’accord avec le moyen que tu choisis? Qui, entre vous deux, prend la décision concernant le type de contraception utilisé? Et penses-tu qu’il pourrait assumer une part de cette responsabilité, par exemple en utilisant le préservatif si certains moyens contraceptifs ne peuvent pas être utilisés pour des raisons de santé?”

**3. Utilisation actuelle de la contraception et comportements à l’égard des méthodes de contraception**

- Quelle méthode contraceptive utilisez-vous actuellement, et pourquoi avezvous fait ce choix?
- Comment décririez-vous votre expérience avec les méthodes contraceptives que vous avez utilisées dans le passé?
- Quelles sont vos principales préoccupations ou réticences par rapport à l’utilisation des contraceptifs?

**4. La taille et le concept de famille**

- Pouvez-vous me parler un peu de ce que le terme “famille” signifie pour vous….
- A quoi pensez-vous quand vous entendez le terme “famille”?
- Pouvez-vous me dire qui fait partie de votre famille?
- Pouvez-vous me parler des différents rôles des membres de la famille? (Par exemple, que fait votre mari pour la famille? Que faites-vous pour votre famille?)
- Comment se prennent les décisions dans votre famille? (Par exemple, comment l’argent est-il dépensé, à quoi sert-il, ou même où vivez-vous et avec qui vivez-vous?)
- Comment définissez-vous la taille idéale d’une famille? Pourquoi?
- Quels facteurs influencent votre choix quant au nombre d’enfants que vous souhaitez avoir?
- En quoi vos expériences personnelles ou culturelles influencent-elles votre vision de la famille idéale?

**5. Connaissances des femmes en matière de planification familiale et de méthodes contraceptives**

- Quelles méthodes de contraception connaissez-vous actuellement?
- Où avez-vous appris les informations que vous possédez sur la planification familiale?
- Selon vous, quelles informations devraient être plus accessibles ou mieux diffusées concernant la contraception?
- Pourriez-vous me décrire comment vous utilisez chaque méthode de contraception? Et que faites-vous en cas d’oubli (exemple de la pilule)?

**6. Barrières affectant l’utilisation des contraceptifs, en particulier le dispositif intra utérin**

- Avez-vous déjà envisagé l’utilisation d’un stérilet (DIU)? Pourquoi ou pourquoi pas?
- Quels obstacles ou difficultés avez-vous rencontrés (ou pensez-vous rencontrer) concernant l’accès aux contraceptifs?
- Selon vous, quels sont les principaux freins socioculturels ou personnels à l’utilisation des méthodes contraceptives dans votre communauté?
- Avez-vous déjà parler avec ton mari sur ce moyen de contraception?
- Quel est son point de vue sur cette ce moyen contraceptif (DIU)
- Avez-vous besoin de son consentement pour utiliser le DIU? quel est son point de vue?
