# Supplementary material for: Family size control in Morocco: a qualitative exploratory study among rural women
Source: Med Trop Sante Int. 2025 Nov 17;5(4):mtsi.v5i4.2025.667. [Article in French] doi: 10.48327/mtsi.v5i4.2025.667 (PMC12948356; doi:10.48327/mtsi.v5i4.2025.667)
Supplement: Related texts

# Research article: Family size control in Morocco: a qualitative exploratory study among rural women

## Introduction

Family planning programs (FPP) that promote contraceptive use have helped reduce fertility rates in many developing countries [13]. Morocco launched its FPP program in 1966, significantly increasing contraceptive coverage and improving maternal and child health [13]. Contraceptive use is a key indicator of successful family planning implementation. It is essential for preventing unwanted pregnancies, spacing births, reducing infant mortality, and limiting unsafe abortions [4]. Over the past two decades, contraceptive use among Moroccan women has increased significantly. Prevalence rose from 9% in 1980 to 63% in 2004 and then to 67.4% in 2011 [13]. However, according to the 2018 National Population and Family Health Survey in Morocco, only 6.8% of women used intrauterine devices (IUDs) as a contraceptive method, despite the effectiveness and reversibility of this method. However, IUD use varied by region, with higher rates in urban areas (8.8%) than in rural areas (3.8%). Of the women who used IUDs, 43% reported concerns, primarily about the potential health effects (32.6%), impact on menstruation (9.4%), and husbands’ disapproval (13.1%) [14].

Significant efforts have been made at the national level to improve access to contraceptives, raise awareness of family planning, and reduce barriers related to cultural norms and women’s knowledge. Previous studies have identified several factors that influence contraceptive use in Morocco. For instance, a study in the Marrakech province found that the number of living children and infant mortality were key determinants of modern contraceptive use. Age at marriage and the age of the woman were also significant factors [16,22]. Furthermore, qualitative research has shown that gender norms such as parental refusal, stigma, and the social exclusion of girls from sexual and reproductive health education services affect access to these services in the Marrakech-Safi region. Family members’ decision-making power regarding contraceptive use is another factor that affects access to these services in the region [16].

These findings underscore the need to strengthen efforts to improve women’s use of contraceptives in Morocco. They also underscore the importance of deepening the understanding of the barriers to adopting certain methods, particularly the IUD, which remains one of the most effective contraceptive options available. In comparison, 47% of married Algerian women of reproductive age who had never used contraception cited opposition from their husbands/partners (12%) and pressure from their partners (4%) as reasons for not using it. Traditional religious and cultural norms, as well as legal obligations, often place men in charge of their wives and families [2].

The main objective of this study was to examine the knowledge, attitudes, and practices of married women of reproductive age living in rural areas of Morocco regarding family planning and the currently available contraceptive methods. Additionally, we aimed to examine the impact of gender norms on family planning decisions and to identify factors that promote or hinder the adoption of contraceptives, particularly long-acting methods such as IUDs.

## Materials and methods

Data were collected in the maternity wards of four provincial hospitals located in different districts of Morocco:

- Hassan II Provincial Hospital in Settat (northwest)
- Moulay Abdellah Provincial Hospital in Salé (northwest)
- Sidi Mohamed Ben Abdellah Hospital in Essaouira (southwest)
- Ibno Baja Hospital in Taza (northeast)

These hospitals serve urban and rural areas in their respective provinces, ensuring diverse representation among the study participants.

The study employed an ethnographic approach to examine Moroccan women’s understanding of family planning, their attitudes toward contraceptive methods, and the obstacles they encounter in accessing contraception. Data were collected through semi-structured interviews with open-ended questions, following an iterative analysis process.

The study targeted married women of childbearing age who had recently given birth, resided in rural areas of the selected provinces, and faced socioeconomic constraints, such as low levels of education and limited access to health services. Participants were recruited during their postpartum stay in the maternity wards from among all those who met the inclusion criteria and consented to participate in the study.

A non-probability sampling strategy combining convenience and judgment sampling was used in the initial phase [5,11,17]. A preliminary analysis of the initial interviews confirmed the appropriateness of the participants and refined the selection criteria. This process resulted in a final sample size of 49 individuals. Data saturation determined the final sample size, and recruitment ceased when no new themes emerged from the participants’ narratives.

Data collection took place from September 2020 to April 2021, spanning eight months. The research process began with a pilot test of the in-depth interview guide with women who shared similar characteristics. This allowed us to refine the research questions and identify terminological gaps. Throughout the study, the in-depth interviews were shaped by the participants’ reproductive narratives, fostering rapport and engagement between the researchers and the interviewees. Some participants requested follow-up interviews, resulting in additional meetings. Interviews lasted between 30 minutes and two hours, averaging one hour and 30 minutes.

The in-depth interviews were conducted using a semi-structured guide developed based on a literature review of the social determinants of family planning and reproductive health in Maghrebi countries and qualitative research tools used in similar studies. The authors then adapted the guide to the local context through discussion, taking into account the cultural and linguistic specificities of women living in rural Morocco.

The guide consisted mainly of open-ended questions designed to examine knowledge, attitudes, and practices related to family planning. The guide also studied the influence of gender norms and the barriers and factors that promote the adoption of contraception (see Appendix 1). This allowed participants to share their experiences in their own words. All interviews were audio-recorded and transcribed for analysis.

The audio recordings were transcribed, and the verbatim transcripts were translated from Moroccan dialect into English. Thematic analysis was used, beginning with generating initial codes, which were then grouped into potential themes. The researchers reread the transcripts several times to refine the emerging themes. Data coding and organization were performed using MAXQDA 2020 software, supplemented by manual analysis in the initial phase.

The study received ethical approval from the Ethics Committee for Biomedical Research at the Faculty of Medicine and Pharmacy in Rabat (CERB: 45/21). Participants provided informed consent prior to participating in the study, thereby ensuring their voluntary participation and confidentiality.

## Results

Table I summarizes the sociodemographic characteristics of the women who participated in the study. Our qualitative data highlight five significant themes related to the participants’ knowledge, attitudes, and practices regarding family planning:

- decision-making regarding family planning;
- current contraceptive use and attitudes toward contraceptive methods;
- women’s family size and concept of family;
- women’s knowledge of family planning and contraceptive methods;
- barriers to contraceptive use, particularly IUDs.

**Table I:** Characteristics of participants

Province	Number of individual interviews	Age group (in years)	Average number of children	Number of housewives	Level of education
Taza	12	(24-44)	3	12	No schooling = 4 Primary = 6 Middle school = 1 High school = 1
Essaouira	13	(19-37)	3	13	No schooling= 4 Primary = 8 Middle school = 1
Salé	11	(20-38)	2,81	10	No schooling= 2 Primary = 4 Middle school = 1 High school = 4
Settat	13	(17-36)	2,53	12	No schooling = 4 Primary = 4 Middle school = 4 High school = 1

Gender plays a crucial role in determining who has the final say in a couple’s use of contraception.

Some women experience additional pressure from their husbands, especially when trying to conceive a son. One interviewee shared her experience: *“I already have four daughters, and my husband wants a son. I can’t take it anymore. In addition to the difficulties I experienced during pregnancy and childbirth due to my cardiovascular disease, my whole family is eagerly awaiting a son.”* (Aicha, Essaouira).

For most people interviewed, decisions about birth control are a joint matter. The choice of contraceptive method often requires the husband’s approval, especially beyond oral contraceptives. Sometimes, this extends to strong resistance to using IUDs. As one participant explained: *“He wanted a third child, but I refused because I didn’t want to get pregnant again. I suggested using an IUD, but he refused because he wants more children*.” (Marzouka, Taza). Data on decision-making regarding family planning and modern contraception show that husbands and in-laws significantly influence when, how, and whether family planning and contraception are used.

Additionally, mothers-in-law wield considerable authority over birth control. They influence their son’s wife, and when they want to expand the family, they often persuade the woman not to use contraceptives and may deny her access to them at health centers. One woman said: “My *husband and his family prefer male children and want to expand the family. However, I have no desire to have more children”* (Randa, Taza).

Another participant said: “My *spouse and his family strongly favor sons. Now that I have had a tubal ligation, they are upset, but that was after I had five children*” (Marzouka, Taza).

The women emphasized that they often resort to family planning due to the personal and physical consequences of pregnancy. “I *don’t want to have any more children. I am satisfied with my current children. I endured significant hardship during pregnancy, which affected my health and left me exhausted”* (Randa, Taza).

Almost all participants used at least one form of contraception, with the exception of primiparous women who adhered to the normative belief that the primary purpose of marriage was procreation and that pregnancy should follow quickly after marriage. Although contraceptive methods are legally available to all married women, those who have never had children rarely use them. None of the new mothers had used modern contraceptive methods to regulate their fertility before the birth of their first child.

The contraceptive pill was the most widely used method among our participants. Multiparous women, in particular, had experimented with various methods. Some complained of side effects and claimed that these methods posed health risks. One participant shared her experience: “I *use the pill to space out my pregnancies, but it has had side effects. I developed a fibroid because of the pill, which led to two abortions”* (Rachida, Taza).

Despite the side effects and risk of forgetting to take it, all the women expressed confidence in the pill. For women who wanted several children, the conventional method was periodic abstinence, raising questions about the need for modern contraception. Another participant said: “I *have been using the pill for five years, since the birth of my first child. It is effective, and I trust this method. I am apprehensive about minor surgical procedures, such as the IUD. People often talk about them negatively*” (Ghizlan, Essaouira).

Although few women chose the IUD as a contraceptive method, those who did were successful and expressed a desire to continue using it for birth spacing or limitation. They were motivated by the positive experiences of their mothers, sisters, and friends. One participant said: “I *recently had an IUD inserted. I used it for seven years before having this child and had it removed to conceive. I plan to return to this method because I found that the pill negatively affected my hormonal balance. With the IUD, I could carry heavy objects without experiencing any side effects. Its effectiveness depends on the condition of the woman’s uterus. A woman with a healthy uterus can perform household chores and lift heavy objects without any problems. My brother-in-law’s wife wore an IUD for 12 years”* (Aicha, Essaouira).

All participants expressed a desire to limit their families to two children of different sexes. Some mentioned that their ideal number was three children.

However, most women with more than two children said they exceeded this ideal because they wanted a girl and a boy, or because of pressure from their husbands or families. Another important factor in having more children is the preference for sons. One participant said: “My *husband wants a son, and I already have two daughters. But I don’t want to have more children because I had difficulties during pregnancy and childbirth. Men often prefer to have a son*” (Kawtar, Salé).

Several women emphasized the importance of having a son in the household for the education of their daughters. They believed that a son would protect his sisters from external threats, accompany them to school, and ensure their safety in their father’s absence. One participant explained: “My *mother-in-law wants a large family. She prays for five daughters or six sons. My husband thinks it is better to have a son first because he can help educate his sisters. A daughter should be the first to make her own decisions, free from her brother’s authority. In our community, when a boy is born first, he often has authority over his sisters. My older brother can do whatever he wants”* (Khadija, Settat). In this context, some of those interviewed preferred to have a boy as their last child in order to spare their daughters from double pressure: one from their father and the other from their brother.

When asked about having more children or considering the issue of fertility and parenthood, respondents often emphasized the increasing complexity of modern life. “I *don’t want to have any more children. Raising children is more demanding nowadays. You have to provide for them financially, which is not easy. Today, children ask us why we brought them into the world if we can’t provide for them”* (Rachida, Essaouira).

The women interviewed revealed that this generation needs substantial financial resources, yet the current financial situation often permits raising only one or two children. They emphasized the physical and financial constraints of having children, which can sometimes exceed their means. One participant said: “*My husband wants four children, but our socioeconomic situation does not allow it. To raise children, you need money, education, and appropriate clothing. It’s not just about having a child; it’s about making sure they have food and clothes. Whether we have two or three children, they must have a good life, appropriate clothing, and an education” (Nezha, Taza).*

The concept of family varies from participant to participant. For most women, family is considered the cornerstone of society, and both men and women aspire to start a family. *“Family is everyone’s destiny. Everyone wants to start a family, and not having children is challenging. One day, I will rely on my children to take care of me when I am old”* (Hafida, Essaouira).

For these women, a family consists of a mother, a father, and at least one child. Parents are responsible for ensuring their children’s well-being, clothing, and education. The father is responsible for providing security, sustenance, and housing for the family, while the mother takes care of her children and husband. Women reported that children obeyed their parents’ instructions and attended school. Some also said that children were considered an investment and that they were expected to provide financial support to their parents as they grew up and repay the care they received during their childhood. One participant explained: “We *bring children into the world to educate them. When they grow up, they must support us financially and stand by our side”* (Es Zahra, Taza).

Women in all regions were able to name several family planning methods, including some modern methods. However, their knowledge of how each method works, its effectiveness, and how to use it was limited. Apart from the most well-known methods, such as pills and injectable contraceptives, participants knew little about IUDs, tubal ligation, implants, and vasectomy. They also mentioned breastfeeding as a traditional approach to preventing pregnancy.

Participants from all regions emphasized the importance of family planning for maternal and child health, as well as for the overall well-being of the family.

Nearly all of the study participants had experienced at least one unwanted pregnancy, primarily due to the incorrect use of contraceptives, particularly the pill; the husband’s inability to abstain during the periodic abstinence method, or family pressure to have multiple children. In some cases, husbands are involved in managing contraception. For example, one participant explained: *“My husband caused an unwanted pregnancy because he gave me a microprogestin pill instead of an estrogen-progestin pill. I took it and got pregnant…” (Fatiha, Salé).*

However, it is concerning that women have limited knowledge about the effectiveness of contraception, particularly the pill. This includes how to take it, how to use it correctly, and what to do if a dose is missed. Almost all women have experienced at least one unwanted pregnancy due to misuse of the pill. This is often attributed to a lack of information about this widely used contraceptive method in Morocco.

One participant said: *“I stopped using the pill when my husband was away. When he returned, I started taking it again the day before he arrived, but I still got pregnant. I think I may not have taken it correctly. I am aware of contraceptive injections, but I am hesitant to use them because of the risk of osteoporosis. I am also apprehensive about the IUD because it could catch my husband’s penis during intercourse”* (Bouchra, Essaouira). In all regions, most women consider family and friends to be the most reliable and accessible sources of information about contraception. They are strongly influenced by their family and friends’ experiences, often adopting a contraceptive method after hearing about a positive experience from a family member, particularly for methods such as the IUD and the pill.

One participant expressed this influence, stating: *“Even if I don’t use them, I know about contraceptive methods such as the ‘Amalia Sghira’ IUD (minor surgery). I have heard from women who have used it that it is effective and beneficial. My sister has had it for seven years, and I am considering using it after my next delivery. My neighbors mentioned that a woman got pregnant with the IUD, but that was because she was carrying heavy objects. I also know a little about injections*” (Samira, Essaouira).

Few women knew that the IUD was a method of contraception. The local term *“Amalia Sghira”,* which means “minor surgery,” evoked negative impressions and concerns about the method because many people mistakenly believed it to be a surgical procedure.

One participant expressed her apprehension, saying: “I *know about Amalia Sghira, but I’m afraid because I don’t understand it very well. I thought it required scissors and surgical equipment.” The term ‘minor surgery’ scared me. A family member told me about her ‘minor surgery,’ which scared me because I thought it was a surgical procedure. I want to space out my pregnancies and wait five years before having a second child*” (Khadija, Settat).

Another woman shared her experience: “I *used ‘Amalia Sghira for three years, but I had it removed because it caused excessive bleeding. Because of it, I couldn’t fast for the entire month of Ramadan”* (Fatiha, Taza).

Myths surrounding the IUD were mentioned as barriers to its use. One myth is the belief that the IUD can “move around in the abdomen, requiring surgery.” Even when their partners consider using the IUD, most women worry that it will limit their ability to do household chores. One woman said, *“The IUD can migrate to the back or an artery. A family member told me that she started having back pain and suspected that the IUD had moved there, so she had it removed*” (Ghizlan, Essaouira).

Some women also mentioned their husbands’ insensitivity to the potential impact of the pill on their health. Husbands often refuse to use condoms because they fear it will affect their virility. Family planning is generally perceived as a female responsibility. *“He refused to use condoms, saying it was my responsibility as a woman to manage family planning,”* said Kawtar from Settat.

Indifference from healthcare providers, long wait times at health facilities, and the unavailability of certain services, such as IUD insertion at primary healthcare facilities, discourage women from seeking contraceptive advice.

Strict restrictions on women’s mobility emerged as a significant barrier in all regions. Women cannot leave their homes without the consent of their husbands or mothers-in-law. Those who venture out alone, even for medical reasons, are considered disrespectful to their families.

One participant described her experience: “*My husband brought me the pill from the pharmacy, and I am not allowed to leave the house without his permission. I ended up getting pregnant. This specific pill, a progestin-only pill, is the reason for my pregnancy. At first, I resisted because he was changing my prescription. The health center where I could get the right pill is far from our home, and arranging transportation and other logistics is difficult. In addition, the infrastructure is unreliable, which further complicates matters”* (Fatiha, Sale).

## Discussion

Improving reproductive health in Morocco has long been a challenge [15], and family planning offers a way to achieve this goal. However, little research has been conducted on the knowledge, attitudes, and practices of Moroccan women regarding family planning [10].

Participants’ narratives demonstrate the significant influence of societal norms, family expectations, and personal experiences on family planning understanding and decision-making in Morocco. These findings are consistent with recent research conducted in other low-income countries [7,8,20]. The results suggest that women’s reproductive autonomy, cultural perceptions of reproduction, and social network pressures interact to influence contraceptive use, sometimes facilitating and sometimes constraining it. These findings corroborate the conclusions of research conducted in other low-income countries [18]. In Morocco, contraceptive use before the first conception is not considered normal, leading women and couples to consider family planning after the *postpartum* period.

The desire to start a family after marriage was a common theme among all participants. This highlights the widespread social belief that a successful partnership involves having children. This notion is closely linked to the importance of motherhood as a means for women to fulfill their destiny and secure a prominent role in the societal construction of idealized femininity [21]. These dominant cultural norms strongly influence our participants’ decisions about family planning after marriage. However, the actual discussions and negotiations took place within the context of their marital partnership.

The absence of male children in the household often leads to an increase in the total number of births, which helps explain variations in family size. From the women’s perspective, men prefer to have sons among their offspring. This implies that, in their view, sons are the family’s protectors in the absence of the father. This finding is comparable to a qualitative study conducted in Ethiopia, which also revealed a preference for boys over girls. Additionally, having a large family is a status symbol and mark of popularity in other communities [8].

In our study, women expressed a preference for small families, primarily citing socioeconomic constraints. This finding aligns with previous studies, which have shown a gradual reduction in the ideal family size in Morocco and similar contexts through national and comparative surveys. For instance, the desired family size in Morocco fell from 5 to 3.7 children between 1980 and 1995, while a significant decline in fertility was observed in rural areas (from 6.6 to 3 children per woman), which was connected to women’s education and employment [3,6]. These results confirm that the preference for smaller families is part of a broader process of demographic transition and sociocultural transformation. However, it is not uncommon for husbands to want larger families. As with other reproductive decisions, women’s choices regarding family size are the subject of ongoing discussions with their husbands and sometimes with their mothers-in-law, who play an important role in Moroccan family dynamics.

Most of the women interviewed in our survey, especially first-time mothers or those who have never used biomedical contraception, had limited knowledge of contraceptive methods. The pill was the most popular choice. Similarly, their understanding of the lactation amenorrhea method was limited, as other recent research has shown [21].

Our study shows that a significant number of women are at risk of unwanted pregnancies due to the misuse of contraceptives. Our findings reveal several myths and barriers associated with various contraceptive methods, particularly the IUD. Many women have misconceptions and concerns about this method based mainly on information from friends, family members, and spouses. Personal experiences and social network anecdotes influence perceptions of safety more than medical advice. Similar results have been reported in other studies, which found that acceptance of the IUD was facilitated by comprehensive advice from healthcare providers, but hindered by fear and rejection from friends, family, and neighbors [9,18].

According to our study’s results, most women want contraceptives that are easy to use and offer freedom in choosing, using, and accessing the most appropriate method without being limited by socioeconomic factors, such as difficulty accessing health services or needing permission from a spouse.

A lack of communication between health personnel and women in family planning units at health facilities, as well as a lack of specific contraceptive options, particularly IUD insertion, in rural areas negatively impact women’s awareness of and access to birth control methods. Therefore, it is imperative to address these issues by providing comprehensive information, encouraging a friendly approach, organizing counseling sessions, and involving husbands to dispel false rumors about contraceptives.

Several interventions have proven effective in similar contexts to address these challenges [1,12,19,23].

- **Community awareness programs**, such as systematic motivational home visits (SMHV), have helped strengthen knowledge of and acceptance of contraception in rural communities. These programs involve community leaders and adapt messages to local realities, contributing to a better understanding and use of contraceptive methods.
- **Strengthening women’s empowerment** is a key factor in improving reproductive health. Programs that strengthen women’s skills, promote their participation in decision-making processes, and improve their access to education and employment have shown positive results in terms of reproductive health.
- **Improving access to reproductive health services**. Expanding reproductive health services, particularly through training community health providers and using telemedicine, has improved access to contraceptive services in remote areas. These approaches facilitate the dissemination of information and access to contraceptive methods.
- **Despite cultural barriers, comprehensive sexuality education** (CSE) has proven effective in reducing risky behaviors and increasing contraceptive use. Integrating CSE into school and community programs, along with teacher training and parental involvement, is essential to its success.

Therefore, an integrated approach combining community awareness, women’s empowerment, improved access to reproductive health services, and CSE is crucial to addressing unmet contraceptive needs and improving the reproductive health of women in rural Morocco.

## Conclusion

Our study highlights the barriers that various gender norms pose to the use of certain contraceptive methods. The family planning decisions of the women in our study are influenced by relevant gender stereotypes.

Our findings reveal that these decisions are influenced by several sociocultural and economic factors, including the husband’s decision-making power, the need for his consent, the preference for male offspring, the inferior status of women, their limited knowledge of contraception, and the influence of the extended family. Despite these constraints, most women desire a safe, reversible method of contraception. Socioeconomic status appears to be a determining factor; couples aspire to a better quality of life for their children and associate birth control with this prospect. However, in disadvantaged households, reducing the number of children remains difficult to envision.

These interconnected aspects give rise to complex considerations that often result in conflicts or divergent preferences among married couples regarding family planning. Women’s participation in decision-making is often limited. due to male authority in the household and related circumstances many women adopt their husbands’ preferences.

To address these issues, it is imperative to dispel misconceptions and fears surrounding IUDs and to improve health professionals’ supportive behavior. Therefore, interventions are needed to strengthen women’s autonomy and bargaining power in family planning decisions.

Prioritizing the availability of treatments such as injectable contraceptives and permanent contraception is essential, as these require partner acceptance. Educating girls and involving men in family planning can facilitate long-term reproductive health decisions and improve women’s understanding of different contraceptive methods.

## Institutional ethics committee statement

The study was conducted in accordance with the Declaration of Helsinki and was approved by the Ethics Committee of the Faculty of Medicine and Pharmacy in Rabat, Morocco (CERB: 45/21).

## Informed consent statement

Informed consent was obtained from all participants involved in the study.

## Funding

This research received no external funding.

## Authors’ contributions

Conceptualization: CM and JETJ; Methodology: CM, JETJ, and LA; Formal analysis: CM and JETJ; Investigation: CM; Data curation: CM and LA; Original draft writing and preparation: CM, SY, and JETJ; Writing and editing: CM, SY, JETJ, and LA; Visualization: JETJ and LA; Supervision: LA. All authors have read and approved the published version of the manuscript.

## Declaration of interests

The authors declare no conflicts of interest related to this work.
